# Simultaneous prediction of transcription factor binding sites in a group of prokaryotic genomes

**DOI:** 10.1186/1471-2105-11-397

**Published:** 2010-07-23

**Authors:** Shaoqiang Zhang, Shan Li, Phuc T Pham, Zhengchang Su

**Affiliations:** 1 Department of Bioinformatics and Genomics, Center for Bioinformatics Research, the University of North Carolina at Charlotte, Charlotte, NC 28223, USA; 2 College of Computer and Information Engineering, Tianjin Normal University, Tianjin 300387, China

## Abstract

**Background:**

Our current understanding of transcription factor binding sites (TFBSs) in sequenced prokaryotic genomes is very limited due to the lack of an accurate and efficient computational method for the prediction of TFBSs at a genome scale. In an attempt to change this situation, we have recently developed a comparative genomics based algorithm called GLECLUBS for *de novo *genome-wide prediction of TFBSs in a target genome. Although GLECLUBS has achieved rather high prediction accuracy of TFBSs in a target genome, it is still not efficient enough to be applied to all the sequenced prokaryotic genomes.

**Results:**

Here, we designed a new algorithm based on GLECLUBS called extended GLECLUBS (eGLECLUBS) for simultaneous prediction of TFBSs in a group of related prokaryotic genomes. When tested on a group of *γ*-proteobacterial genomes including *E. coli *K12, a group of firmicutes genomes including *B. subtilis *and a group of cyanobacterial genomes using the same parameter settings, eGLECLUBS predicts more than 82% of known TFBSs in extracted inter-operonic sequences in both *E. coli *K12 and *B. subtilis*. Because each genome in a group is equally treated, it is highly likely that similar prediction accuracy has been achieved for each genome in the group.

**Conclusions:**

We have developed a new algorithm for genome-wide *de novo *prediction of TFBSs in a group of related prokaryotic genomes. The algorithm has achieved the same level of accuracy and robustness as its predecessor GLECLUBS, but can work on dozens of genomes at the same time.

## Background

With the continuous decline in the cost of genome sequencing due to the development of new technologies [1,2], numerous prokaryotic genomes are being sequenced, and this number will soon approach a few thousand. Since the biological functions of an organism are encoded in its genome, knowing its genome sequence can greatly facilitate the understanding of its biological functions. However, due to the expensive nature of experimental characterization of biological functions of an organism, ideally, these functions should be largely deduced computationally from its genome sequence. Nevertheless, understanding the function of even a relatively simple prokaryotic cell from its genome sequence remains one of the most daunting challenges in the post-genomic era. In particular, we know very little about the *cis*-regulatory elements or transcription factor (TF) binding sites (TFBSs) in the vast majority of sequenced prokaryotic genomes because of the lack of an accurate and efficient computational method for predicting TFBSs in sequenced genomes.

The difficulty of computational prediction of TFBSs in a prokaryotic genome is mainly due to the short length and degenerate nature of TFBSs, which complicates their discovery within the long upstream inter-operonic regions in which they usually reside. Furthermore, although some TFBSs in prokaryotes have a palindromic structure, any segment of an inter-operonic sequence can in principle be a TFBS as long as a TF can recognize it. Therefore, TFBSs are usually predicted by comparative analysis of multiple sequences that are known to contain or potentially contain TFBSs. Based on the observation that the transcriptional regulation machinery including TFBSs is relatively conserved in closely related genomes, various forms of *phylogenetic footprinting *algorithms have been developed to identify conserved DNA segments as possible TFBSs in the promoters of orthologous genes in a group of related prokaryotic [3-9] and fungal genomes [10]. For the convenience of discussion, in this paper, we refer a set of similar TFBSs as a motif.

These algorithms typically start by predicting TFBSs in the upstream intergenic sequences of a group of orthologous genes using a motif-finding tool, and then cluster the resulting motifs into distinct sets according to the similarity values among the motifs using different clustering strategies and similarity measures [3-10]. Although meaningful results have been achieved by these algorithms in their specific applications, their prediction coverage of possible TFBSs in the applied genomes is generally low [3-10]. For instance, using a Bayesian clustering algorithm to group similar TFBSs predicted in *E. coli *K12 by phylogenetic footprinting in an earlier work [3], Qin *et al. *[4] could only predict 192 motifs covering only 438 operons, while the *E. coli *K12 genome is predicted to encode 266-314 TFs, and more than 2000 operons [11-14]. In another study, van Nimwegen *et al. *[5] used a Monte Carlo sampling strategy to partition into clusters a set of TFBSs predicted by phylogenetic footprinting [3]; this study yielded only 115 significant clusters/motifs. More recently, Liu *et al. *[8] used the PhyloNet algorithm [15] to cluster putative TFBSs predicted by the motif-finding program CONSENSUS-v6c [16] through phylogenetic footprinting in the *Shewanella oneidensis *genome, finding that PhyloNet is not able to efficiently cluster the predicted TFBSs. Therefore, an additional hierarchical clustering procedure was used to achieve reasonable predictions [8].

In our opinion, there are two unnecessarily exclusive problems in these existing algorithms, limiting their performance and applications. First, these algorithms used only a single motif-finding tool in the phylogenetic footprinting process for identifying putative TFBSs. However, it has been shown that these motif-finding tools can only predict at most 30% of known TFBSs in the input intergenic sequences, and may be biased to some types of TFBSs, though different tools may complement with each other for recovering different types of TFBSs [17,18]. Second, most of these algorithms assume that the putative TFBSs predicted by a phylogenetic footprinting procedure are all true TFBSs; therefore the subsequent clustering procedure is designed to group similar motifs into distinct ones without filtering out the spurious predictions. However, a considerable portion of the predicted motifs are clearly spurious predictions due to the low prediction accuracy of current motif-finding tools [17,18].

To overcome these problems, we have recently developed a new algorithm named GLECLUBS (GLobal Ensemble and Clustering of Binding Sites) for genome-wide *de novo *prediction of TFBSs in a prokaryotic genome [19]. Although GLECLUBS also employs a phylogenetic footprinting technique to first identify all possible TFBSs, and then clusters similar motifs, it is distinct from the prior methods in two ways. First, in order to harvest as many as possible true TFBSs by phylogenetic footprinting, GLECLUBS uses multiple well-evaluated complementary motif-finding tools instead of using only a single tool, and considers multiple outputs of each tool. Second, GLECLUBS assumes that only a small portion of predicted TFBSs by phylogenetic footprinting are true TFBSs, and that the vast majority of them are spurious predictions. Therefore, the goal of the clustering step of GLECLUBS is to discriminate true TFBSs from spurious ones using an iterative filtering procedure, instead of simply partitioning putative TFBSs into distinct groups. We have shown that GLECLUBS outperforms the existing algorithms in terms of the prediction sensitivity and specificity in *E. coli *K12, *B. subtilis *and *S. oneidensis *[19]. We found that the major bottleneck for the prediction accuracy of GLECLUBS is the accuracy of operon predictions that are used to guide the extraction of inter-operonic sequences for phylogenetic footprinting [19]. When inter-operonic sequences are correctly extracted, GLECLUBS can recover at least 80% known TFBSs in both *E. coli *K12 and *B. subtilis*, according to RegulonDB [20] and DBTBS [21], respectively.

Nevertheless, GLECLUBS and all of the other prior algorithms that we are aware of, only aim at predicting TFBSs in a single target genome even though multiple reference genomes are used during the phylogenetic footprinting and subsequent clustering steps; therefore the information derived for the TFBSs in the reference genomes are not fully utilized. For this reason, all these algorithms including GLECLUBS are not efficient enough to be applied to all the sequenced prokaryotic genomes. In this study, we have developed a new version of GLECLUBS called extended-GLECLUBS (eGLECLUBS) for simultaneous *de novo *prediction of TFBSs in a group of prokaryotic genomes that we call a group of target genomes. We showed that eGLECLUBS can achieve at least the same level of prediction accuracy for a group of genomes as GLECLUBS does for a single genome; however, it can simultaneously predict TFBSs in dozens of closely related genomes. Therefore eGLECLUBS is more efficient than GLECLUBS, and can be used for predicting TFBSs in the increasing number of sequenced prokaryotic genomes.

## Results

### TFBSs can be effectively identified by phylogenetic footprinting based on predicted COORs using multiple motif-finding tools

**Figure 1:**
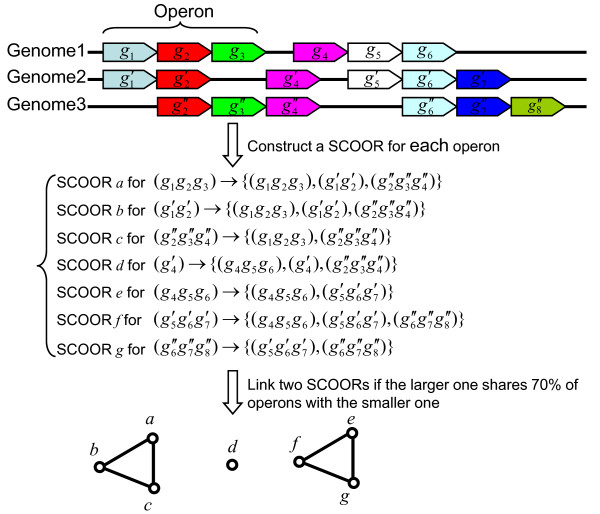
**Schematic show of the algorithm for the prediction of COORs**. Orthologous genes are represented by arrowed boxes of the same color. For each operon *o *in each genome, we construct a semi-COOR (SCOOR) by recruiting *o *and all other operons in other genomes if such an operon contains orthologous genes of > 50% of genes in *o*. We connect any two SCOORs if the larger one contains > 70% of operons in the smaller one. We predict each connected component in the resulting graph to be a COOR.

**Figure 2:**
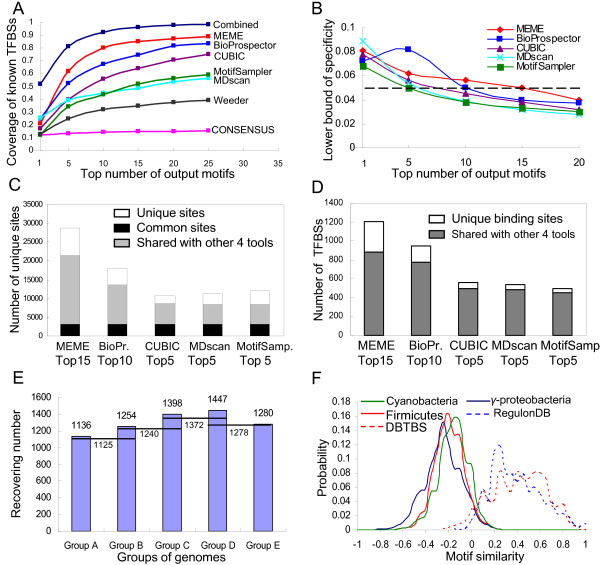
**Selection of parameters of the algorithm**. (A). Performance of motif-finding tools returning different numbers of top predictions for recovering known TFBSs in *E. coli *K12 in the inter-operonic sequence sets. (B). Low bound prediction specificity of the motif-finding tools returning different numbers of top predictions for recovering known TFBSs in *E. coli *K12 in the inter-operonic sequence sets. (C). The number of total sites that are uniquely predicted by a tool or jointly predicted by the tool and other four tools. (D). The number of known TFBSs in *E. coli *K12 that are uniquely predicted by a tool or jointly predicted by the tool and other four tools. (E). Effect of the selection of different groups of target genomes on the prediction of known TFBSs in *E. coli *K12. (F). The distribution of motif similarity scores among the input motifs in the target genomes and that of motif similarity scores among the sub-motifs of the known motifs in *E. coli *K12 (RegulonDB) and *B. subtilis *(DBTBS).

In a typical phylogenetic footprinting procedure with a single target genome, upstream intergenic sequences are extracted based on a group of orthologous genes of a gene in the target genome [3-10]. We [19] have previously shown that intergenic sequences extracted from a group of orthologous operons determined by an operon in the target genome outperforms intergenic sequences extracted based on a group of orthologous genes determined by a gene in the target genome for motif-finding. In current study, in order to predict TFBSs in a group of genomes, we predict *Clusters of Operons with Orthologous Relationships *(COORs, Figure 1 and see Methods) in the genomes. We then extract upstream inter-operonic sequences based on the predicted COORs. Clearly, by the design of the algorithm for predicting COORs, the extracted sequences are unlikely biased to any genome in the group. Application of the algorithm (Figure 1) to a group of target genomes comprised of 32 *γ*-proteobacterial genomes including *E. coli *K12 [Additional file 1: group D in Supplemental Figure S1] resulted in 4,103 COORs and inter-operonic sequences sets which contain 1,447 known *E. coli *K12 TFBSs as described above. To evaluate whether or not we can effectively identify these known *E. coli *K12 TFBSs in the inter-operonic sequence sets based on the COORs, we applied seven motif-finding tools that we have evaluated previously [19] to these 4,103 inter-operonic sequence sets. These seven tools were MEME [22], BioProspector [23], MotifSampler [24], CUBIC [25], MDScan [26], Weeder [27] and CONSENSUS [28]. These tools were chosen based on evaluations by others [17,18], the balance of different algorithm designs and ease of use. As shown in Figure 2A, these tools have different performances in their ability to recover known *E. coli *K12 TFBSs in the inter-operonic sequence sets for their best, top 5, 10, 15, 20 and 25 predictions, but they all identify an increasing number of known *E. coli *K12 TFBSs when more predictions are considered. We can define a lower bound of specificity as the number of predicted known TFBS divided by the number of predicted TFBSs, to evaluate the prediction specificity of each tool, although since TFBs in *E. coli *K12 have not been completely characterized, this estimate of specificity may be overly conservative. As shown in Figure 2B, the increasing number of known *E. coli *K12 TFBSs recovered by each tool when more top predictions are considered is at the cost of a decreased lower bound of specificity. Furthermore, the predictions of these tools are complementary to one another as their combined predictions recover more known TFBSs than does any single tool (Figure 2A). However, Weeder and CONSENSUS substantially underperform the other tools, and their predictions were all covered by the other tools, therefore they were not further considered. Using a low bound specificity cutoff of 5% (Figure 2B), and based on the results shown in Figure 2A, we consider for further analysis a total of 40 motifs in each inter-operonic sequence set associated with a COOR, including the top 15 predictions of MEME, the top 10 predictions of BioProspector, and the top 5 predictions of CUBIC, MDscan and MotifSampler, respectively. Therefore, there are a total of 4,103 × 40 = 164,120 predicted motifs (called input motifs, see Methods) for this group of target genomes. These predicted motifs recover 1,347 (1347/1447 = 94%) known *E. coli *K12 TFBSs in the extracted inter-operonic sequence sets. As shown in Figures 2C and 2D, although there are large overlaps among the predictions of these tools, each tool has its own considerable unique predictions. Therefore, we have achieved at least the same level of sensitivity as our previous results obtained from the inter-operonic sequences based on operons using *E. coli *K12 as the single target genome [19]. These results suggest that TFBSs can be effectively identified based on the predicted COORs, and that our choice of these five tools and their top predictions are sufficient enough to recover true TFBSs in the inter-operonic sequence sets, though other choices of motif-finding tools are possible, in particular, when better ones are available in the future. Furthermore, although our predictions were only evaluated in *E. coli *K12 because very little is known about TFBSs in other genomes in the group, it is highly likely that similar results have been achieved for the other genomes in the group since the COORs are unlikely biased to any genome in the group (see Methods).

### Optimal selection of a group of target genomes is vital for the prediction of TFBSs

Since each genome in a group of target genomes is both a target genome for which we want to predict all possible TFBSs and a reference genome for all the others in the group, the composition of genomes in a group can largely affect the results of phylogenetic footprinting. Furthermore, it has been shown that *cis*-regulatory systems in prokaryotes evolve very rapidly and are extremely flexible [29], thus traditional phylogenetic analyses may fail to detect conserved *cis*-regulatory systems. To quantify the level of conservation of *cis*-regulatory systems in a group of related genomes, we constructed a special phylogenetic tree that largely reflects the relationships of the *cis*-regulatory systems in the genomes, using a method that we developed previously[19] (see Methods). The tree for 139 *γ*-proteobacterial genomes is shown in [Additional file 1: Supplemental Figure S1]. To determine the level of conservation of *cis*-regulatory systems, so that phylogenetic footprinting would perform best in the corresponding genomes for recovering known TFBSs, we selected five sub-trees including *E. coli *K12 using different branching points with increasing distances in the tree for the *γ*-proteobacterial genomes [Additional file 1: Figure S1], and obtained five groups of genomes as follows: group A containing *Escherichia *genomes, group B containing *Escherichia *and *Shigella *genomes, group C containing *Escherichia*, *Shigella*, and *Salmonella *genomes, group D containing *Escherichia*, *Shigella*, *Salmonella*, and *Yersinia *genomes, and group E containing *Escherichia*, *Shigella*, *Salmonella*, *Yersinia*, *Pseudomonas*, *Shewanella*, and *Vibrio *genomes. As shown in Figure 2E, groups A and B did not perform very well because only genomes that are very closely related to each other were used. Similarly, group E did not perform well either, presumably because some genomes in the groups have *cis*-regulatory systems that are too divergent. In contrast, groups C and D that include genomes encoding *cis-*regulatory systems that are intermediately related to that of *E. coli *K12 are the best in recovering the known *E. coli *K12 TFBSs in the extracted intergenic sequences. Interestingly, removal of other very closely related *Escherichia *genomes in groups C and D decreased the performance of phylogenetic footprinting (data not shown). This result is consistent with an earlier observation that inclusion of reference genomes that are very closely related to the target genome may improve the performance of phylogenetic footprinting if other reference genomes that are intermediately related to the target genome are also included [30]. As shown in Figure 2E group D recovers the largest number of known *E. coli *K12 TFBSs and has the largest number of overlapping predictions with those of its neighboring groups C and E. Since group D does not include any genomes that share less than 50% of TFs with any other genomes, we used this as the criterion for selecting a group of target genomes for the prediction of their TFBSs. In other words, by default, we select a sub-tree from the phylogenetic tree of *cis*-regulatory systems of a group of sequenced genomes belonging to the same phylum/subphylum or class to form a group of target genomes, such that each genome in the sub-tree shares at least 50% of its TFs with any other genomes in the group (see Methods). Accordingly, we used group D to evaluate the performance of our motif clustering algorithm.

### **Selection of the motif similarity cut-offs *α *and *β *for the construction of motif similarity graphs G_1 _and G_2_, respectively**

**Figure 3:**
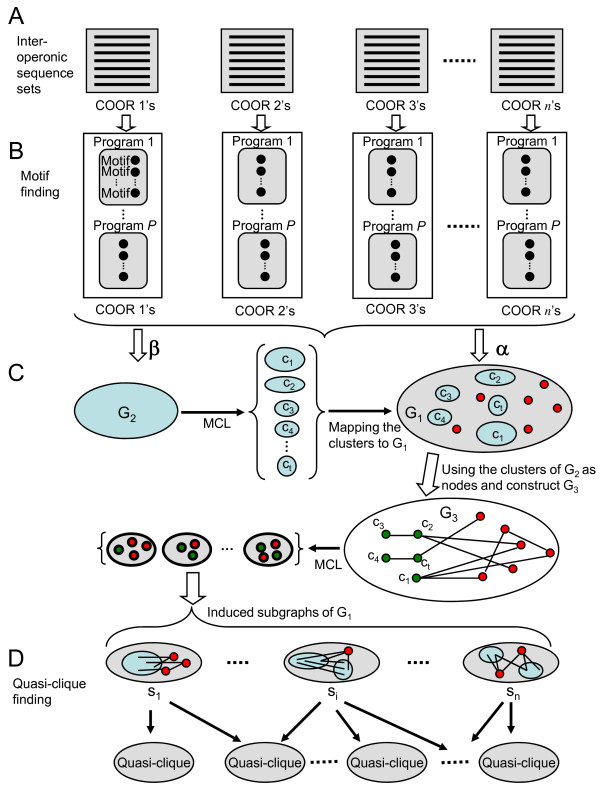
**Flowchart of the main steps of the eGLECLUBS algorithm**. (A). We extract upstream inter-operonic sequences of operons in each *COOR *to form a set of sequences. (B). For each sequence set, we predict a total of *T *motifs using multiple motif-finding tools. (C). We construct two motif similarity graphs *G*_1 _and *G*_2 _using a low and a high motif similarity score cut-offs *α *and *β*, respectively, and cut *G*_2 _into dense subgraphs using MCL. We construct *G*_3 _and cluster it into dense subgraphs using MCL. (D). We induce subgraphs *s*_1_, *s*_2_, ..., *s_n _*of *G*_1 _using the clusters from *G*_3_, and identify quasi-cliques in each of these induced subgraphs.

**Figure 4:**
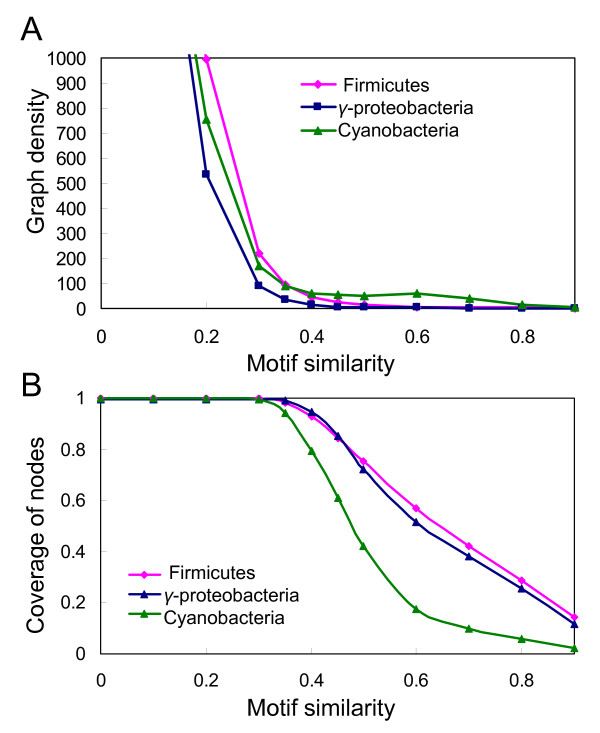
**Selection of motif similarity score cutoffs for the construction of motif similarity graphs**. (A). Graph density as a function of motif similarity cut-off. (B). The number of nodes in a motif similarity graph as a function of motif similarity cut-off.

Next, we want to distinguish true TFBSs from spurious ones in the set of input motifs predicted for a group of target genomes by gradually filtering out the latter based on the following two assumptions as we used in GLECLUBS previously [19]: 1) a true motif is more likely than a spurious one to be found by multiple tools from the same inter-operonic sequence set based on a COOR; and 2) a true motif is more likely than a spurious one to have a similar motif found in a different COOR. To this end, we use a graph-theoretic approach similar to that used in GLECLUBS. However, instead of first constructing a single motif similarity graph, we begin with two motif similarity graphs *G*_1 _and *G*_2 _using a low and a high motif similarity score cut-offs *α *and *β *(*α *<*β*), respectively (Figure 3, and see Methods). As shown in Figure 2F, the distribution of the similarity scores among all predicted motifs in the group is largely left-shifted relative to that among the sub-motifs of each known motif in *E. coli *K12, suggesting that the majority of predicted motifs are irrelevant to one another or are spurious predictions. On the other hand, there is a considerable overlap between the two distributions of the similarity scores, suggesting that the true motifs cannot be easily separated from the spurious ones by a single similarity score cut-off. The same conclusion was reached by us previously when *E. coli *K12 was chosen as the single target genome [19]. Furthermore, in the current application we have to deal with a much large set of input motifs (1.6×10^5^) identified from the 4,103 inter-operonic sequence sets based on the same number of COORs in the group of genomes, which is almost twice more than those when *E. coli *K12 was used as the single target genome [19]. Ideally, a motif similarity graph should be constructed, so that most relevant true sub-motifs are connected by edges with higher weights, while most irrelevant and spurious motifs are not. According to the similarity score distributions shown in Figure 2F, the optimal similarity score cut-off value seems to be located around 0.05. However, we found that with this similarity score cut-off or lower, the density of a resulting graph (defined as the number of its edges divided by the number of its nodes) was too high (more than 10^3^). And a graph of this size (>10^5 ^nodes) could not be efficiently clustered into dense subgraphs/clusters by any clustering algorithms that we have tested, including the Markov chain clustering (MCL) algorithm [31] that is best known for its high efficiency for clustering very large graphs. However, the density of the motif similarity graphs decreases precipitously when the motif similarity cut-off is greater than 0.2 (Figure 4A) while the resulting graphs still include the vast majority (99.9%) of input motifs as long as the cut-off is less than 0.3 (Figure 4B). Therefore, we selected the cut-off *α *∈[0.2, 0.3] for the construction of motif similarity graph *G*_1 _that includes the vast majority of input motifs. Nevertheless, *G*_1 _usually still has a too high density (>500 when *α *= 0.2, Figure 4A) to be efficiently clustered, therefore, we construct a substitute motif similarity graph *G*_2 _using a higher motif similarity score cut-off *β *∈[0.35, 0.45]. Since *G*_2 _has a low enough density (<100), thus can be efficiently clustered by the MCL algorithm, while the graph still keeps more than 90% of the input motifs (Figure 4B). Therefore, our clustering algorithm works by first clustering *G*_2 _into dense subgraphs, and then recruits those in *G*_1 _but missed in *G*_2 _(see Methods).

### Prediction of TFBSs in a group of *γ*-proteobacterial genomes including *E. coli *K12 and a group of firmicutes genomes including *B. subtilis*

The eGLECLUBS algorithm ranks the predicted motifs in each genome in a group of target genomes according their *ClusterScores *defined by formula (4). To evaluate the prediction accuracy and robustness of our algorithm, we first applied it to a group of 32 *γ*-proteobacterial genomes including *E. coli *K12 [Additional file 1: group D in Supplemental Figure S1], and a group of 19 firmicutes genomes including *B. subtilis *[Additional file 1: Supplemental Figure S2] using the same parameter settings. These two groups of genomes were chosen for the evaluation as the relatively large numbers of known TFBSs in *E. coli *K12 and *B. subtilis *can be used to systematically verify our predictions in the two genomes.

As shown in Table 1, of the 1,642 and 568 known TFBSs belonging to 125 and 99 motifs in *E. coli *K12 [20] and *B. subtilis *[21], 1,447 and 451 belonging to123 and 93 motifs, respectively, are correctly extracted and included in the upstream inter-operonic sequence sets according to the predicted operons in the genomes [32,33] and our criterion that each set has to contain at least three sequences (see Methods). Consequently, about 12 and 21% of TFBSs in *E. coli *K12 and *B. subtilis *[32,33], respectively, were not included in the inter-operonic sequence sets due to the incorrect operon predictions or the restriction of our criterion, and thus could not be predicted by our clustering algorithm. The reason that there are a larger portion (21%) of missed TFBSs in the extracted sequence sets for *B. subtilis *than that (12%) for *E. coli *K12 is that more inter-operonic sequence sets containing a sequence from *B. subtilis *were deleted as the corresponding COORs contain fewer than three operons, and thus were not further analyzed (see Methods). The five motif-finding tools returning a total of 40 motifs in each inter-operonic sequence set recover 1,347 and 397 known TFBSs belonging to 122 and 92 motifs in *E. coli *K12 and *B. subtilis*, respectively (Table 1). Thus, 93 and 88% of known TFBSs included in the inter-operonic sequence sets are recovered by the phylogenetic footprinting procedure in *E. coli *K12 and *B. subtilis *[32,33], respectively (Table 1).

**Table 1:** Recovery of known TFBSs and motifs in each step of the eGLECLUBS algorithm for *E. coli *K12 and *B. subtilis*

Genomes	Motifs/TFBSs	RegulonDB/DBTBS	COORs	Phylogenetic footprinting	Clustering*
*E. coli *K12	TFBSs	1642	1447 (88%)	1347 (93%)	1102(82%)
	
	Motifs	125	123 (98%)	122 (99%)	113 (93%)

*B. subtilis*	TFBSs	568	451 (79%)	397 (88%)	324(82%)
	
	Motifs	99	93 (94%)	92 (99%)	88 (96%)

The percentage in a brace is the recovery rate at that step based on the previous step. *The calculations are based on the top 300 and 230 motifs/clusters predicted in *E. coli *K12 and *B. subtilis*, respectively.

**Figure 5:**
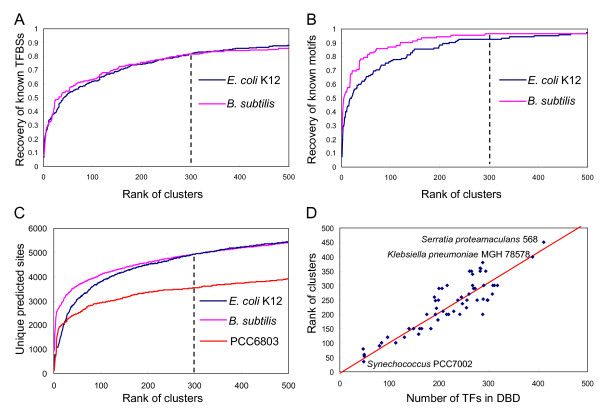
**Evaluation of the predictions in the groups of target genomes**. (A). Cumulative recovery rate of known TFBSs in the input motifs by the top-ranked clusters. (B). Cumulative recovery rate of known motifs in the input motifs by the top-ranked clusters. (C) Cumulative number of unique predicted TFBSs in the top-ranked clusters. (D) The rank of the cluster at which the cumulative number of unique predicted TFBSs becomes saturated in a genome is highly correlated to the number of predicted TFs in the genome according to the DBD database.

We then calculated the cumulative recovery rate by the top-ranked motifs of the 1,347 and 397 known TFBSs found by phylogenetic footprinting in *E. coli *K12 and *B. subtilis*, respectively. As shown in Figure 5A, with the increase in the number of top-ranked clusters, the cumulative recovery rate of known TFBSs by the top-ranked clusters increases very rapidly for the top 300 and 230 clusters, which recover 1,102 (82%) and 324 (82%) of the 1347 and 397 known TFBSs in *E. coli *K12 and *B. subtilis*, respectively, and then it enters a saturation phase with a small linear increase. We also calculated the recovery rate of the 122 and 92 motifs by the top-ranked clusters in *E. coli *K12 and *B. subtilis*, respectively. We consider that a known motif is recovered by one cluster if at least 20% of its known TFBSs are included in this cluster. As shown in Figure 5B, with the increase in the rank of clusters, the cumulative recovery rate of known motifs by the top-ranked clusters increases rapidly for the top 300 and 230 clusters, recovering 113 (93%) and 88 (96%) of the 122 and 92 motifs in *E. coli *K12 and *B. subtilis*, respectively, and then it also enters a saturation phase with little increase. Therefore, our clustering algorithm has achieved rather high sensitivity in recovering the known TFBSs as well as the known motifs of both genomes. Interestingly, the cumulative recovery rate of known motifs saturate at around the top 300-th and 230-th predicted motifs, which is close to the number of TFs that have been estimated in the *E. coli *K12 [11-14] and *B. subtilis *genomes [34], respectively. The rapid recovery of known TFBSs (Figure 5A) and motifs (Figure 5B) by top-ranked clusters also suggest that the higher the rank of a cluster, the more likely it is a true motif.

To assess the specificity of the predictions in *E. coli *K12 and *B. subtilis*, we calculated the number of cumulative unique predicted TFBSs in the top-ranked clusters. We consider a predicted TFBS to be unique if there is no other site overlapping with it by more than eight bases. As shown in Figure 5C, with the increase in the rank of clusters, the number of cumulative unique predicted TFBSs in both genomes increases in a way very similar to the cumulative recovery rates of known TFBSs shown in Figure 5A, and then it saturates around the top 300-th and 230-th clusters, covering about 4,900 and 4,600 unique TFBSs in *E. coli *K12 and *B. subtilis*, respectively.

There are 40,405 and 57,567 unique predicted TFBSs in the whole set of 164,120 and 18,6960 input motifs predicted by phylogenetic footprinting in *E. coli *K12 and *B. subtilis*, respectively. Therefore, the vast majority of predicted TFBSs have been filtered out by our algorithm in both genomes. The fact that these relatively small numbers (4,900 and 4,600) of unique predicted TFBSs recover 1,102 (82%) and 324 (82%) of 1,347 and 397 known TFBSs in the top 300 and 230 clusters of *E. coli *K12 and *B. subtilis*, respectively, suggests that our clustering algorithm has significantly enriched the true TFBSs in the predictions (*p *< 10^-10 ^according to a hyper-geometric distribution for both genomes). It is highly likely that our algorithm has achieved high prediction specificity in both genomes, although we cannot accurately compute the prediction specificity and false positive rate, because not all TFBSs in both genomes are currently known. However, we can estimate the low bound of prediction specificity based on the partially known TFBSs to be 22% (1102/4900) and 7% (324/4600) by the top 300 and 230 clusters in which 113 (113/300 = 30%) and 88 (88/230 = 38%) motifs recovered in *E. coli *K12 and *B. subtilis*, respectively. As shown in [Additional File 1: Supplemental Tables S1 and S2], the top 20 predicted motifs in both *E. coli *K12 and *B. subtilis *recover 10 known motifs, achieving a low bound specificity of 50% in both genomes. Therefore, we have achieved at least the same level of prediction accuracy in both genomes as we have previously obtained when *E. col*i K12 and *B. subtilis *were used as the single target genome [19].

In the current study since our selection of genomes in the two groups of target genomes, and prediction of COORs are not biased to any particular genome, e.g., *E. coli *K12 or *B. subtilis *in the groups, it is highly likely that we have also achieved the same level of prediction accuracy for all of the genomes in the groups, although we cannot systematically verify our prediction in the genomes other than *E. coli *K12 or *B. subtilis*, because none of these genomes has enough number of known TFBSs for doing so. However, if we inspect the predictions in any of these less-studied genomes, biologically meaningful results can be always identified http://gleclubs.uncc.edu/pbs. For example, if we look at the top-ranked motifs in *Yersinia pestis *biovar Mediaevails that is relatively far away from *E. coli K12 *in the tree [Additional file 1: Supplemental Figure S1] in the *γ*-proteobacterial group, the 2nd top-scoring motif is similar the CRP binding sites in *E. coli *K12, and the genes that bear a binding site of this motif are annotated to be involved in carbon and energy metabolism, therefore it is highly likely that this is the CRP binding motif in the genome. The 3rd top-scoring motif is similar to the FUR motif in *E. coli *K12, and genes that bear a binding site of this motif are annotated to be involved in ion assimilation, therefore it is highly likely that this motif is recognized by FUR in this genome. The 32-nd top-scoring motif is associated with genes involved in arginine biosynthesis, thus is likely to be the binding motif of the regulator ArgR in this genome. The binding sites of the 43-rd top-scoring motifs are born by genes involved in pentose metabolism, so this motif is likely to be involved in the regulation of pentose metabolism, however, its cognate TF cannot be predicted based on the current data. The 50-th top-scoring motifs is similar to the LexA motif in *E. coli *K12, and many genes bearing a binding site of this motif are annotated to be involved in DNA damage repairing, so it is likely that this motif is recognized by LexA in this genome. Similar results are seen for the other Yersinia genomes in the group http://gleclubs.uncc.edu/pbs. Therefore, eGLECLUBS can not only predict known TFBSs in model organisms, but also can predict novel TFBSs in less-studied organisms. The predicted TFBSs in the 32 *γ*-proteobacterial and 19 firmicutes genomes can be found at the website of eGLECLUBS http://gleclubs.uncc.edu/pbs. More importantly, with only one more graph clustering step than its predecessor GLECLUBS (see Methods), eGLECLUBS can predict TFBSs in a group of up to 32 genomes. Therefore, eGLECLUBS is more efficient than GLECLUBS.

Furthermore, these results also show that we have achieved the similar prediction accuracy in *B. subtilis *based on the 19 firmicutes genomes as in *E. coli *K12 based on the 39 *γ*-proteobacterial genomes using the same parameter settings, suggesting that our algorithm is very robust in predicting TFBSs in different groups of genomes. In addition, it is noticeable that the cumulative binding sites, motifs and unique putative binding sites in *B. subtilis *all saturate faster than those of *E. coli *K12 (Figure 5), respectively, although both genomes encode similar number of genes (4,105 vs. 4,132) and operons (about 2,300 vs. 2,400). This might indicate that *B. subtilis *encodes fewer TFs than does *E. coli *K12. In fact, according to the DBD database [35], the *E. coli *K12 genome was estimated to encode 266 TFs, while the *B. subtilis *genome 238 TFs.

### Prediction of TFBSs in a group of cyanobacterial genomes

To further evaluate the prediction accuracy and robustness of eGLECLUBS, we applied it to a group of 14 target genomes chosen from 33 sequenced cyanobacteria [Additional file 1: Supplemental Figure S3] using the same parameter settings. Despite the important roles of cyanobacteria in global primary production and the evolution of green plants, our current understanding of *cis-*regulatory systems in even the best-studied cyanobacterial *Synechocystis *sp. PCC 6803 strain (PCC6803) is still very limited. Since there are not many known TFBSs in any cyanobacterial genome, we cannot systematically verify our predictions in a way as we did in *E. coli *K12 and *B. subtilis*. However, as shown in Figure 2F, the similarity scores of the input motifs for this cyanobacterial group have a very similar distribution to those of the *γ*-proteobacterial and firmicutes groups, suggesting that eGLECLUBS should also perform well in this group of genomes. Furthermore, if we use our predictions in PCC6803 as an example, as shown in Figure 5C, the number of cumulative unique putative TFBSs in the top-ranked clusters in the genome rapidly saturates at ~3,354 around the 200-th cluster, indicating that we have likely enriched the true TFBSs in PCC6803 as in the cases of *E. coli *K12 and *B. subtilis*. Again, the much faster saturation of the cumulative unique predicted TFBSs in PCC6803 than in *B. subtilis *and *E. coli *K12 (Figure 5C) suggests that the number of TFs in PCC6803 might be fewer than those in these two genomes. Indeed, DBD predicts that there are 85 TFs in PCC6803 [36], even though the actually number might be greater than this number [36]. Moreover, a few experimentally characterized or computationally predicted motifs by other ad hoc methods are among the top 20 predicted motifs in PCC6803 [Additional file 1: Supplemental Table S3], including the PhoB motif (9-th) [37,38], NtcA motif (11-th) [39,40], and CRP motif (14-th) [41]. It is worth noting that, although the CRP regulons in cyanobacteria are rather diverse as we recently showed [41], eGLECLUBS can still accurately identify the CRP binding motif in PCC6803 with a high rank. Therefore, these results again unequivocally demonstrate that eGLECLUBS is robust enough to predict TFBSs in various groups of less-studied genomes with high accuracy.

### Correlation between the number of predicted motifs and the number of TFs encoded in genomes

As shown in Figure 5D, the number of top-ranked clusters at which the cumulative unique predicted TFBSs saturate is highly correlated to the number of predicted TFs in the DBD database for each genome in the three groups of target genomes. Therefore, the saturation point can be used to roughly estimate the number of motifs encoded in each genome in a group of target genomes. These results again suggest that our predictions of TFBSs in each genome have achieved a high level of accuracy, and that our algorithm is very robust in terms of parameter settings for different groups of target genomes. Interestingly, the two bacteria that have the largest number of predicted motifs and TFs, namely, *Serratia proteamaculans *568 and *Klebsiella pneumoniae *MGH 78578 (Figure 5D), are free-living pathogens of human and animals [42]http://www.ncbi.nlm.nih.gov/genomeprj/31. Their more complex gene regulatory systems might be due to their needs to cope with very different living environments. In contrast, the bacterium that has the smallest number of predicted motifs and TFs is the *Cyanobacterium synechococcus *PCC7002, which thrives in seawater where nutrients are relatively stable. Furthermore, cyanobacteria can acquire their carbon source through photosynthesis, and thus generally have simpler gene regulatory systems.

## Discussion

Phylogenetic footprinting followed by motif clustering has been proven the most practical method for genome-wide TFBS prediction in prokaryotic genomes. However, the existing methods including the GLECLUBS algorithm that we developed earlier [19] were designed to predict TFBSs in a single target genome of interest, and the TFBSs in multiple reference genomes were largely ignored and not predicted. Therefore these algorithms are not very efficient and cannot be applied to a large number of sequenced genomes. The major reason for this practice is that these methods identify orthologous gene groups for phylogenetic footprinting based on the gene contents in the target genome, therefore, inter-operonic sequences pooled based on these orthologous gene groups are inevitably biased to the target genome, and the coverage of predictions in reference genomes may not be high enough to be used for the genome-wide prediction of TFBSs in the genomes. In the current study, we have developed the eGLECLUBS algorithm for simultaneous genome-wide prediction of TFBSs in each genome in a group of genomes without introducing a bias to any genome via overcoming two technical obstacles. First, we identify COORs in a group of target genomes based on the gene contents and operon structures in each genome, and treat each genome in the group equally both as a target genome and as a reference genome for the others. Therefore, the pooled inter-operonic sequences are unlikely to be biased to a particular genome. Second, because we consider every gene in each genome in the group, we end up with about twice as many inter-operonic sequence sets as we have when a single target genome was considered [19]. To circumvent the difficulty of clustering a much larger motif similarity graph with relatively high density for predicting TFBSs, we construct two similarity graphs *G*_1 _and *G*_2 _with a high and a low motif similarity score cut-offs, respectively. We first cut *G*_2 _into dense clusters, and then recruit in the resulting clusters the input motifs in G_1 _but not in *G*_2_, and reconnect those that are not connect in *G*_2 _but in *G*_1_. True motifs are then gradually separated from spurious ones through multiple rounds of subsequent graph clustering. Application of the eGLECLUBS algorithm to a group of 32 *γ*-proteobacterial genomes including *E. coli *K12, a group of 19 firmicutes genomes including *B. subtilis*, and a group of 14 cyanobacterial genomes, resulted in similar prediction results in both the *E. coli *K12 and *B. subtilis *genomes as we have achieved when each was used as the single target genome by GLECLUBS [19]. Due to the limited knowledge of TFBSs in most sequenced genomes, we can only systematically verify our predictions in the two model bacteria *E. coli *K12 and *B. subtilis*. We assert, however, that it is highly likely that we have achieved the same level of prediction accuracy for each genome in the three groups of genomes because of the unbiasedness of our algorithm to any genome in the groups. Therefore, we have improved our original algorithm for simultaneously predicting TFBSs of dozens of genomes without a cost to prediction accuracy. To further speed up the algorithm, we are currently parallelizing the eGLECLUBS algorithm through distributed computing, so that it can be applied to all the sequenced prokaryotic genomes in the public databases.

In addition, with a single set of parameter settings, our algorithm performs equally well on the groups of *γ*-proteobacterial, firmicutes and likely cyanobacterial genomes. Therefore, it is very robust for predicting TFBSs in various groups of genomes. The robustness of our algorithm can be explained by the similar distributions of the similarity scores among the input motifs identified in the different groups of target genomes, and the similar distributions of the sub-motifs of the known motifs in different genomes (Figure 2F). Consequently, the coverage of nodes and the graph density of motif similarity graphs under different motif similarity score cut-offs in the three groups are similar (Figure 4A and 4B). Therefore, the motif similarity score cut-offs used to construct motif similarity graphs for the three groups of genomes work equally well. Since these distributions are determined by the first principles of biochemical rules, thus they are unlikely to be genome-specific. Therefore, we expect that our algorithm can be applied to any group of target genomes using similar parameter settings.

## Conclusions

We have developed a new algorithm for genome-wide *de novo *prediction of TFBSs in a group of related prokaryotic genomes. The algorithm has achieved rather high prediction accuracy, and is very robust and computationally efficient. The resulting tool can be used for annotating TFBSs in the increasing number of sequenced prokaryotic genomes.

## Methods

### Materials

The genome sequences and their annotation files of *γ*-proteobacteria, firmicutes and cyanobacteria were downloaded from the NCBI ftp sever ftp://ftp.ncbi.nih.gov/genomes. The known TFBSs of *E. coli *K12 and *B. subtilis *were downloaded from RegulonDB Version 6.0 [20] and DBTBS Release 5 [21], respectively. Known and predicted TFs were downloaded from DBD Release 2.0 http://www.transcriptionfactor.org[35]. Predicted prokaryotic operons were downloaded from the DOOR database [32,33], which has the highest prediction accuracy among all surveyed operon prediction algorithms [43].

### Selection of a group of target genomes

To select a group of target genomes for which we want to predict TFBSs in each genome, we used a previously developed method that considers not only the evolutionary relationships, but also the number of shared TFs among the genomes in the group [19]. Briefly, we selected all the sequenced genomes from the same phylum or class of interest, e.g. the 139 sequenced *γ*-proteobacterial genomes, 124 sequenced firmicutes genomes, and 33 sequenced cyanobacterial genomes as it has been shown that genomes from the same phylum or class usually share TFBSs that are conserved enough to be predicted by phylogenetic footprinting [3-10]. We then computed a bits vector for each genome, where "1" and "0" represents the presence and absence of a known or predicted TFs in the genome according to the DBD database [35]. We constructed a neighbor-joining tree based on the Hamming distance between the vectors of each pair of genomes. By selecting difference branches, we could obtain different groups of target genomes with different levels of conservation of gene transcriptional regulatory systems.

### Predictions of orthologs and COORs

Orthologous proteins and their genes between two genomes were predicted by the bi-directional best hits (BDBH) method [44] using the BLASTP algorithm with an E-value cut-off 10^-20 ^in both directions of search. For each operon *o *of each genome *G_i _*in a group of target genomes, we construct a group of operons called a *Semi-Cluster of Operons with Orthologous Relationship *(SCOOR) as follows (Figure 1): The SCOOR initially only contains the operon *o*; for each genome except *G_i _*in the group of target genomes, if there exists an operon *o*' containing orthologs of at least 50% of genes in *o*, we recruit *o*' into the SCOOR. Next, we construct a graph using these resulting SCOORs as the set of vertices. We connect two SCOORs by an edge if the larger one includes 70% of the operons in the smaller one. Because of the low connectivity, the graph is composed of many connected components (a connected component is defined as a subgraph in which any two vertices are connected by at least a path.). We call the operons associated with each connected component a *Cluster of Operons with Orthologous Relationships *(COOR). Clearly, in a COOR, the majority of genes in each operon in a genome have orthologs in other operons from other genomes. A COOR is conceptually similar to a cluster of orthologous transcription units defined by Wels et al. [9].

### Prediction of input motifs

For each COOR *C_i _*containing at least three operons, we extracted up to 800 bases upstream inter-operonic sequence for each operon in *C_i _*to form a set ***I***_*i *_of sequences. Therefore each ***I***_*i *_contains at least three inter-operonic sequences, which is necessary for most motif-finding tools to work well. We apply *P *motif-finding tools to each ***I***_*i*_, and each tool *j *returns its top *T_j _*motifs (Figure 3A and 3B). The length of returned motifs is set to be 16 bases for all tools as we found that motif-finding tools perform best with this length in prokaryotes [19]. Therefore, there are *T *= *T*_1 _+ *T*_2 _+ ... + *T*_P _putative motifs for each ***I***_*i*_. If there are *n *COORs containing at least three operons in the groups of target genomes, we will have a total of *nT *motifs, which are referred to as *input motifs*.

### Calculation of motif-motif similarity

We used the same motif similarity metric that we defined previously [19] to compute a similarity score for each pair of input motifs. We have shown that this metric outperforms the other existing ones for separating true motifs from spurious ones. Briefly, for a motif *M*_*x *_containing *n*_*x *_sequences with length *L_x_*, let  be its frequency matrix, and *P_x _*be its profile matrix defined as(1)

where *p_x _*(*b*, *i*) is the probability of base *b *∈{*A*, *C*, *G*, *T*} appearing at position *i *of *M_x_*, and *q*(*b*) is the probability of base *b *appearing in the background sequences. For two motifs *M*_1 _= (*P*_1_, *F*_1_) and *M*_2 _= (*P*_2_, *F*_2_), the similarity score between them is defined as(2)

where(3)

Note that in the metric (3), *A *is the set of optimal ungapped alignments that have the maximum number of aligned columns {*i*}, each satisfying , and column *i *of *P_x _*corresponds to column *s*(*i*) of *F_y _*in the alignment *s *∈ *A*.

### Prediction of TFBSs in a group of target genomes through graph clustering

In our previous design of the GLECLUBS algorithm [19], we constructed a motif similarity graph using the predicted input motifs as the nodes and connecting two nodes with an edge if their similarity scores is above a preset cut-off; we then clustered the graph into dense subgraphs using the MCL algorithm [31]. However, we found that when the size of the graph increases to the size of current application, the MCL algorithm becomes too inefficient to cluster the graph. To circumvent this obstacle, we first construct two motif similarity graphs *G*_1 _= (*V*_1_, *E*_1_) and *G*_2 _= (*V*_1_, *E*_1_) with a high and a low graph densities, respectively (Figure 3). The density of a graph is defined as the number of its edges divided by the number of its nodes in this paper. We choose the density of *G*_1 _to be high enough, so that most relevant motifs are presumably connected, and the density of *G*_2 _to be low enough so that only highly similar motifs are connected and the graph can be efficiently cut by the MCL algorithm. We first cut *G*_2 _into dense subgraphs/clusters, and then recruit in the resulting clusters the motifs not in *G*_2 _but in *G*_1_(Figure 3). The details of the clustering procedure follow.

(A) *Construct motif similarity graphs G*_1 _*with a high density and G*_2 _*with a low density, and cut G*_2 _*into dense subgraphs using MCL*. We construct two motif similarity graphs *G*_1 _= (*V*_1_, *E*_1_) and *G*_2 _= (*V*_2_, *E*_2_) using predicted motifs as the nodes, and connecting any two motifs by an edge if their similarity score is greater than two preset cut-offs *α *and *β *(*β *>*α*), respectively, with the similarity score being the weight on the edge (Figure 3C). Because of the larger value of *β*, *G*_2 _contains fewer nodes and edges than does *G*_1_. We choose a value of *α *so that most relevant motifs are connected in *G*_1_, and a value of *β *so that the density of *G*_2 _is low enough to be efficiently clustered into dense subgraphs using the MCL algorithm. We typically choose *α *and *β *such that the density of *G*_1 _is about 500, and that of *G*_2 _is about 15~20 (see Results). We then apply MCL to *G*_2 _to cut it into dense subgraphs, denoted by *C *= {*c*_1_, *c*_2_, ..., *c_t_*}(Figure 3C).

(B) *Construct and cluster motif similarity graph G*_3_. Let *V*_1 _- *V*_2 _= {*v*_1_, *v*_2_, ..., *v_k_*} be the nodes in graph *G*_1 _but not in *G*_2_. We construct graph *G*_3 _= (*V*_3_, *E*_3_), where the node set *V*_3 _= *C *∪(*V*_1 _- *V*_2_) = {*c*_1_, ..., *c_t_*, *v*_1_, ..., *v_k_*}, and the edge set *E*_3 _is defined as follows. For each pair of nodes *u*, *v *∈ *V*_3 _we calculate , where *w_xy _*is the weight of edge (*x*, *y*) ∈ *E*_1_, and |*u*| and |*v*| the number of nodes of *u *and *v*, respectively. We connect *u *and *v *by an edge (*u*, *v*) ∈ *E*_3 _with a weight  if and only if  is greater than the cut-off *α *used to construct *G*_1_. We then apply MCL to *G*_3 _to obtain a set of clusters (Figure 3C). The motifs in each of these resulting clusters induce a subgraph of *G*_1_. Let these induced subgraphs be the set ***S ***= {*s*_1_,*s*_2_,...,*s_n_*} (Figure 3D).

(C) *Construct and cluster the quasi-clique-based motif similarity graph G*_4_. For each subgraph *s_i _*in ***S***, we apply the method described in our previous work [19] to find a clique associated with each node in *s_i_*, and then merge all cliques into a so-called *quasi-clique *if any two cliques share the majority of their nodes (Figure 3D). For each quasi-clique, we pool all the corresponding putative TFBSs, and merge any two sequences if they overlap more than 8 bases to form a quasi-clique-specific sequence set. We then identify the best motif for each of the quasi-clique-specific sequence sets using the motif-finding tool BioProspector [23]. We call this motif a *quasi-clique-specific motif*. We then calculate the similarity score between each pair of quasi-clique-specific motifs, and construct a new motif similarity graph *G*_4 _using the quasi-clique-specific motifs as the nodes, and connecting any two nodes if the similarity between the two motifs is larger than a preset cut-off *γ *(= *β*, normally). We cluster graph *G*_4 _into a set of dense subgraphs using MCL.

(D) *Construct and cluster the extended sequence-based motif similarity graph G*_5_. For each cluster from *G*_4_, we pool all the corresponding putative binding site sequences, and merge any two sequences from the same intergenic sequence if they overlap more than 8 bases to form a new non-overlapping sequence set. To fix the possible problem of only covering a part of a binding site by motif-finding tools because of the use of a fixed motif length for motif finding so far, we extend each sequence on both ends by padding a fixed length (10 bases) of its flanking genome sequences. For each of these extended sequence sets, we identify the best 22-base long motif called an *extended motif*. Similar to the construction of graph *G*_1_, we construct a new motif similarity graph *G*_5 _using the same motif similarity score cut-off *γ *as used to construct *G*_4_. We then cluster *G*_5 _into a set of subgraphs using MCL.

(E) *Construct and cluster genome-specific motif similarity graphs*. Note that each motif in the clusters from *G*_5 _contains predicted TFBSs from different genomes in the groups of target genomes. We pull the putative binding sites in each cluster from *G*_5 _into a group if they are from the same genome to form a *genome-specific sequence set*. So sequences in each cluster are partitioned into multiple genome-specific sequence sets, and there are multiple genome-specific sequence sets from the same genome. For each genome, after merging the overlapping sequences in each genome-specific sequence set, we find the best motif in each of its genome-specific sequence sets using the motif-finding tool MEME [22] with the length being automatically detected in the region of 8-22 bases. We then calculate motif-motif similarity between each pair of the resulting motifs for the genome, and construct a genome-specific motif similarity graph using the similarity score cut-off *γ *used to construct *G*_4_. The graph is clustered into subgraphs using MCL.

(F). *Refine and rank the clusters of each genome*. The clusters obtained from the genome-specific motif similarity graph contain sequences with different lengths, we identify up to top 15 motifs of different lengths (= 22 - 8 + 1) from the sequences of each cluster using MEME with the motif length being automatically detected in the region of 8~22 bases. Since the resulting motifs may cover different parts of the same motif, and contain the same TFBS because of the high similarity of sequences in each cluster, we merge the overlapping and redundant sequences covered by different motifs to form a set of unique sequences, which is the final predicted motif in each cluster. We use the following scoring function defined previously [19] to evaluate the likelihood that a cluster contains a true motif,(4)

where *n *is the number of sequences in the best motif of length *L *found by MEME, *p*(*b*, *i*) and *P*(*b*, *i*) are the probability and profile, respectively, of base *b *appearing at position *i *of the motif as defined in formula (1), and *N *is the number of sequences in the cluster. We rank all the clusters in descending order according to their *ClusterScore*s.

## Availability

The eGLECLUBS algorithm is currently implemented in Perl, consisting of a set of utility programs for generating input files and a suite of programs for TFBS prediction in genomes. The source codes and detailed instructions for running the programs are available at http://gleclubs.uncc.edu/pbs.

## Abbreviations

GLECLUBS: Global Ensemble CLUsters of Binding Sites; eGLECLUBS: extended GLECLUBS; ORF: open reading frame; TFBS: transcription factor binding site; COOR: Clusters of Operons with Orthologous Relationships; SCOOR: semi-COOR; TF: transcription factor.

## Authors' contributions

ZS conceived the project. SZ designed and conducted the experiment. SL and PTP helped conduct some analysis. ZS and SZ wrote the manuscript. All authors read and approved the final manuscript.

## Supplementary Material

Additional file 1 **Supplementary figures and tables**. Additional file 1 consists of three supplementary figures and tables. Supplemental Figure S1: The phylogenetic tree of *cis*-regulatory systems in sequenced *γ*-proteobacterial genomes for the selection of groups of target genomes containing *E. coli *K12. Supplemental Figure S2: The phylogenetic tree of *cis*-regulatory systems in sequenced firmicutes for the selection of a group of target genomes containing *B. subtilis*. Supplemental Figure S3: The phylogenetic tree of *cis*-regulatory systems in sequenced cyanobacteria for the selection of a group of target genomes containing *Synechocystis *sp. PCC 6803. Supplemental Table S1: The top 20 motifs/clusters predicted in *E. coli *K12. Supplemental Table S2: The top 20 motifs/clusters predicted in *B. Subtilis*. Table S3: The top 20 motifs/clusters predicted in *Synechocystis *sp. PCC 6803. Click here for file
