# Supplementary material for: Simultaneous prediction of transcription factor binding sites in a group of prokaryotic genomes
Source: BMC Bioinformatics. 2010 Jul 23;11:397. doi: 10.1186/1471-2105-11-397 (PMC2920276; doi:10.1186/1471-2105-11-397)
Supplement: Additional file 1 — Supplementary figures and tables. Additional file 1 consists of three supplementary figures and tables. Supplemental Figure S1: The phylogenetic tree of cis-regulatory systems in sequenced γ-proteobacterial genomes for the selection of groups of target genomes containing E. coli K12. Supplemental Figure S2: The phylogenetic tree of cis-regulatory systems in sequenced firmicutes for the selection of a group of target genomes containing B. subtilis. Supplemental Figure S3: The phylogenetic tree of cis-regulatory systems in sequenced cyanobacteria for the selection of a group of target genomes containing Synechocystis sp. PCC 6803. Supplemental Table S1: The top 20 motifs/clusters predicted in E. coli K12. Supplemental Table S2: The top 20 motifs/clusters predicted in B. Subtilis. Table S3: The top 20 motifs/clusters predicted in Synechocystis sp. PCC 6803. [file 1471-2105-11-397-S1.PDF]

Additional File S. Zhang *et al.*, Figure S1

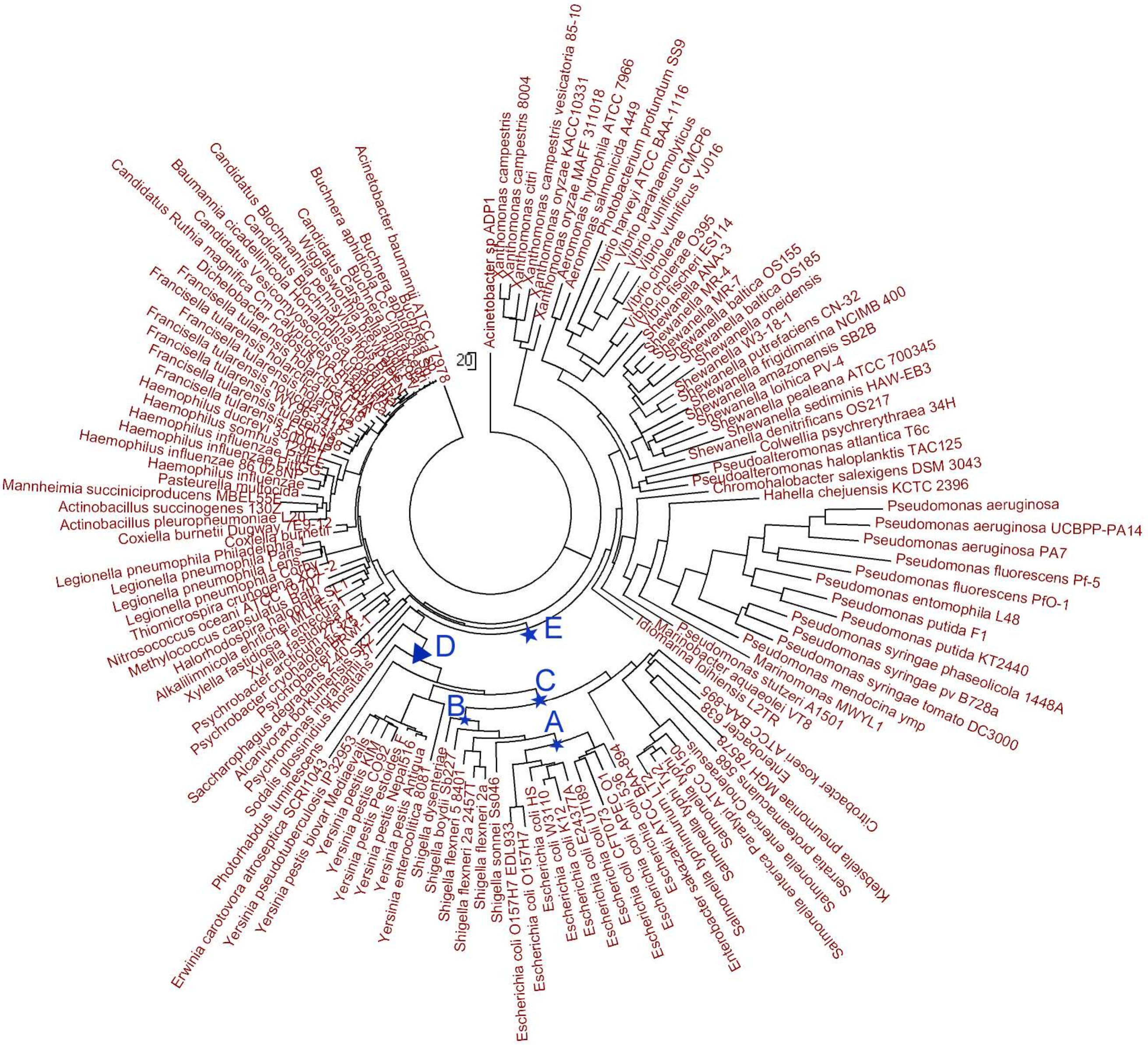

Figure S1: The phylogenetic tree of *cis*-regulatory systems in sequenced γ-proteobacterial genomes for the selection of groups of target genomes containing *E. coli* K12. A labeled arrow indicates the branch points for the selection of a group of target genomes.

S. Zhang *et al.*, Table S1

Rank	Web logo	Structure / Consensus / Covering known motifs	Rank	Web logo	Structure / Consensus / Covering known motifs
1	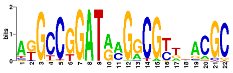		11	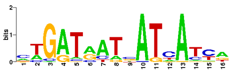	Fur 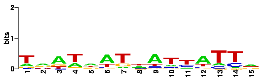
2	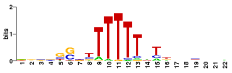	T-rich	12	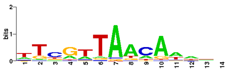	Palindromic TTTGTTAACAAA
3	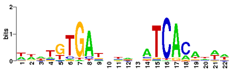	CRP 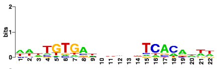 FNR 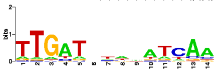	13	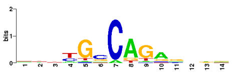	TGnCAGA
4	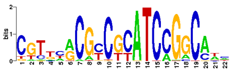		14	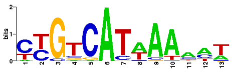	PhoB 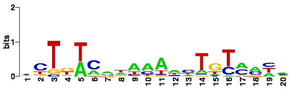
5	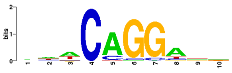		15	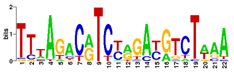	*MetJ* 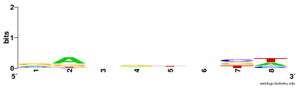
6	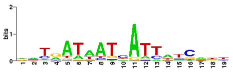	IHF 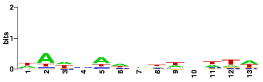	16	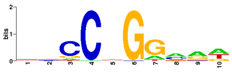	Palindromic CCnGG
7	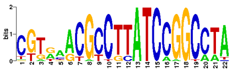	ACGCCTTATCCGGC CTA	17	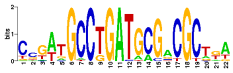	
8	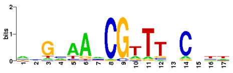	PurR(63%) 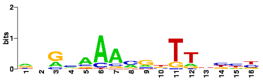	18	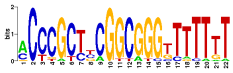	Palindromic ACCCGCtncgGCGGGT
9	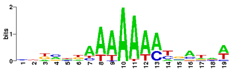	A-rich	19	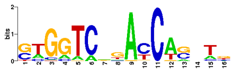	FadR 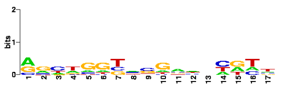
10	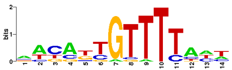	PrpR 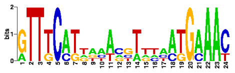	20	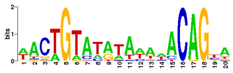	LexA 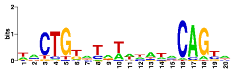

The logo is for the best motif identified by MEME in each cluster.

S. Zhang *et al.*, Figure S2

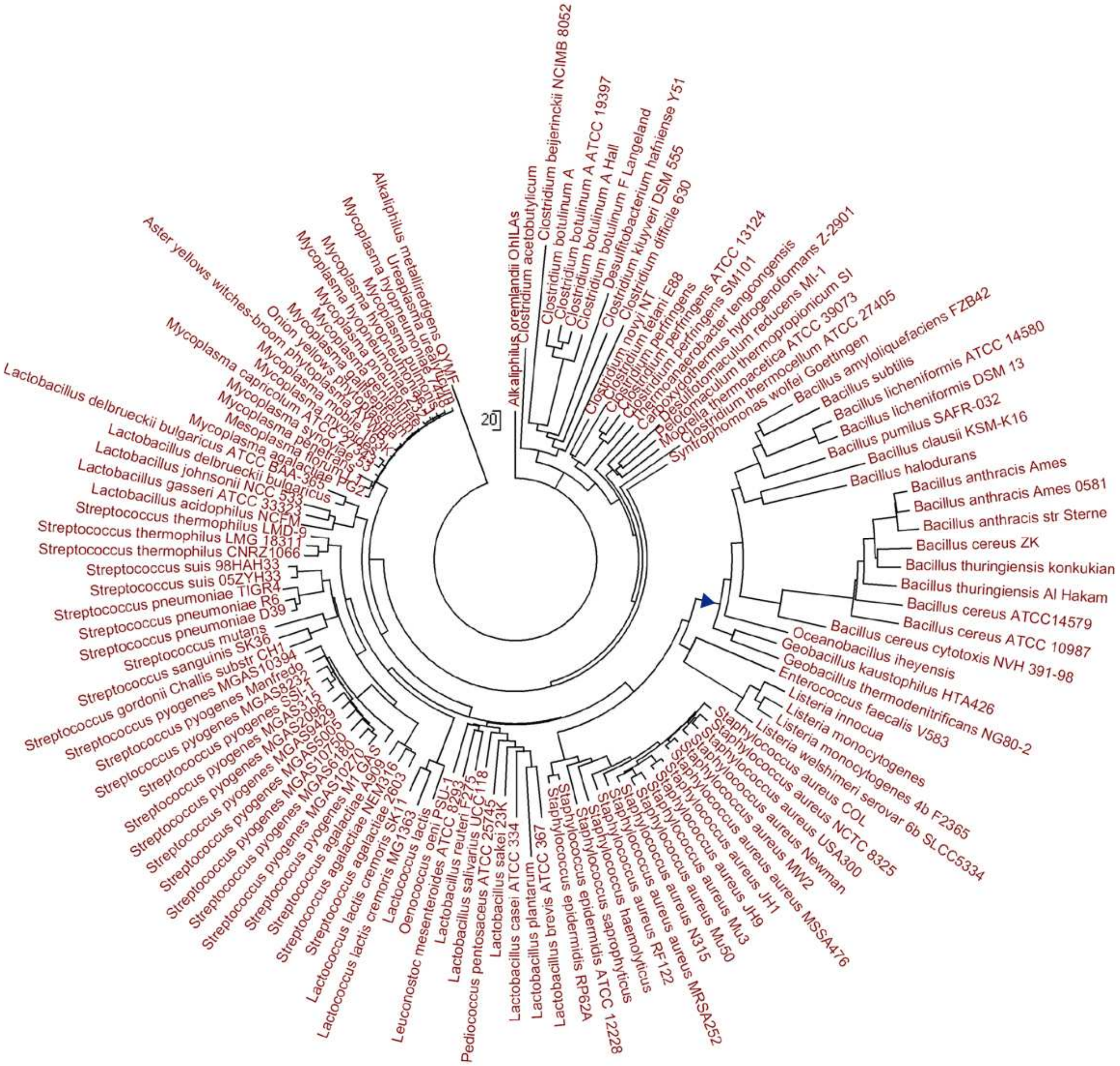

Figure S2: The phylogenetic tree of *cis*-regulatory systems in sequenced firmicutes for the selection of a group of target genomes containing *B. subtilis*. The labeled arrow indicates the branch point for the selection of the group of target genomes.

S. Zhang *et al.*, Table S2

Rank	Web logo	Structure / Consensus / Covering known motifs	Rank	Web logo	Structure / Consensus / Covering known motifs
1	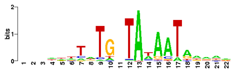	TGnTATAAT (sigma-factor)	11	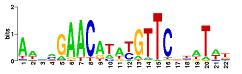	LexA (51%) 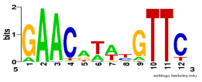
2	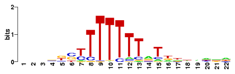	T-rich	12	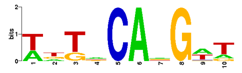	TnTnCAnGnT
3	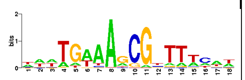	CcpA (33%) 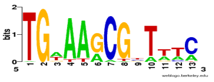	13	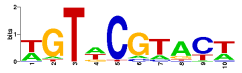	AraR (88%) 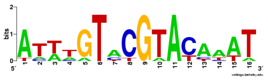
4	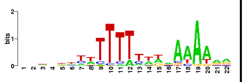	TTTTnnnnAAAA	14	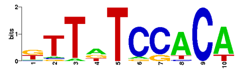	DnaA (90%) 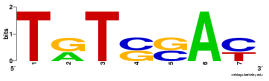
5	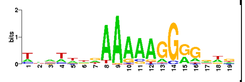	AAAAAGGGG	15	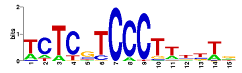	TCTCnTCCCT
6	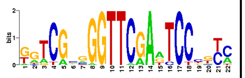	GGTCGnnGGTTCGAnTCC	16	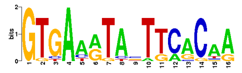	YdiH (86%) 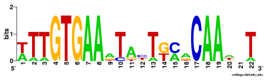
7	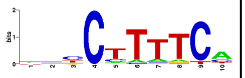	CTTTTC	17	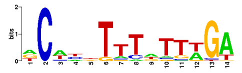	CnnnTTTnTTTGA
8	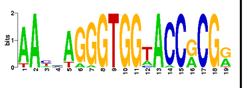	tRNA syntheses (Cover 100% in DBTBS) 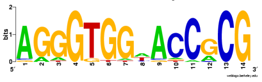	18	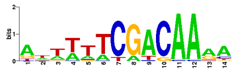	TTTTCGACAAAA
9	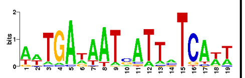	Fur (63%) 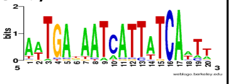 PerR (27%) 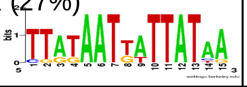	19	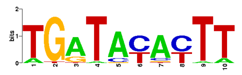	TGATACACTT
10	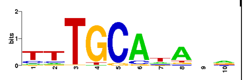	TTTGCAAA	20	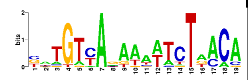	TnrA (30%) 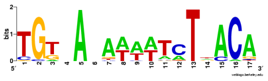

The logo is for the best motif identified by MEME in each cluster.

S. Zhang *et al.*, Figure S3

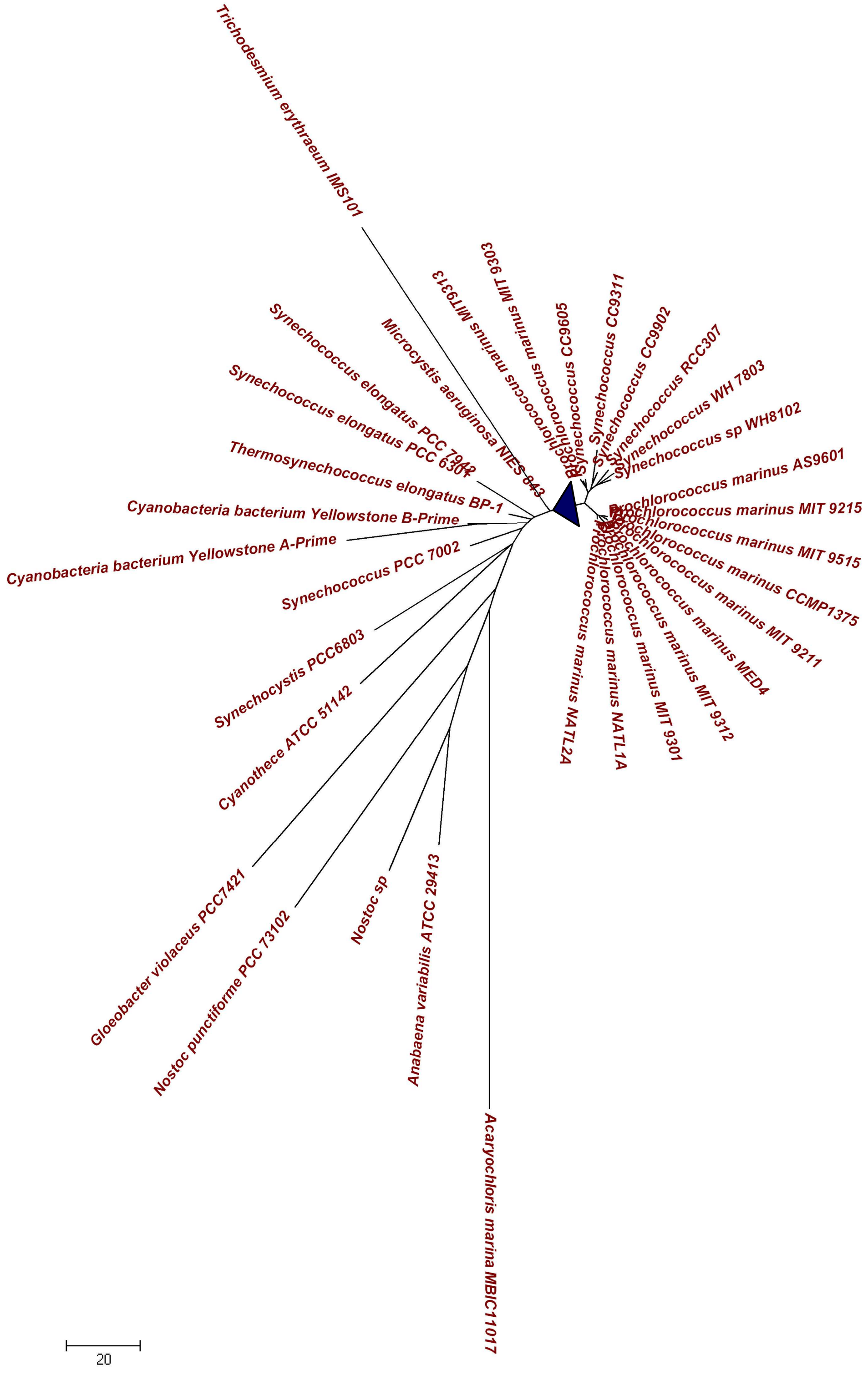

Figure S3: The phylogenetic tree of *cis*-regulatory systems in sequenced cyano-bacteria, which can be naturally parted into two groups of genomes from the triangle.

S. Zhang *et al.*, Table S3

Rank	Weblogo	Structure(or TFs)/ Consensus	Rank	Weblogo	Structure(or TFs)/ Consensus
1	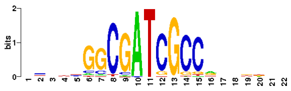	Palindromic GGCGATCGCC	11	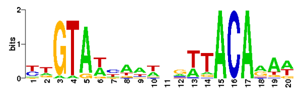	*NtcA* binding sites TGTAnnnnnnnnTA CA
2	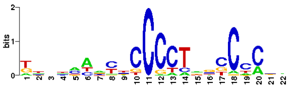	CCCCTnnCCCC	12	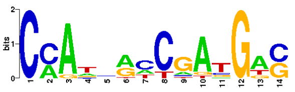	Palindromic CCATnnnnATGA
3	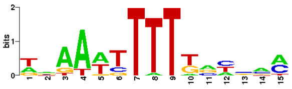	AAATTTT	13	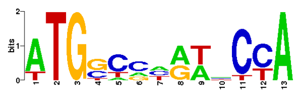	Palindromic TGGnnnnnnCCA
4	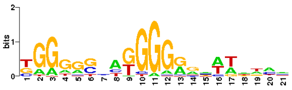	Tandem repeat GGGGGnnGGGG G	14	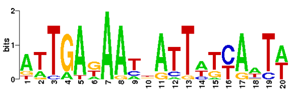	*CRP* binding sites ATTGAnAATnATT nTCAAT
5	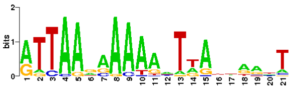	ATTAAnAAAAAnTT A	15	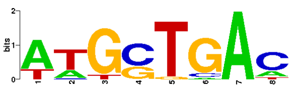	ATGCTGAC
6	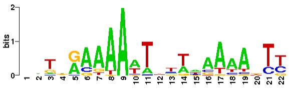	Tandem repeat AAAAnTnnTnAAA A	16	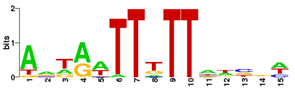	TTnTTn
7	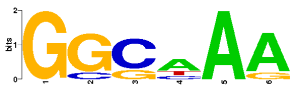	GGnnAA	17	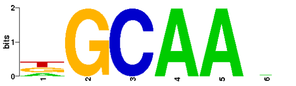	GCAA
8	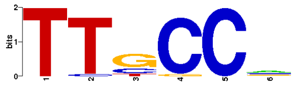	TTnCCn	18	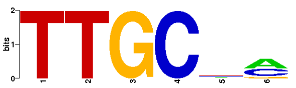	TTGC
9	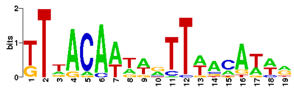	*PhoB* binding sites TTnACAnnnTTnA CA	19	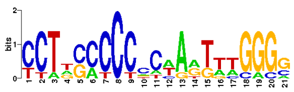	Palindromic CCCCAAATTTGG GG
10	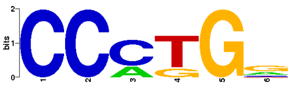	CCCTGG	20	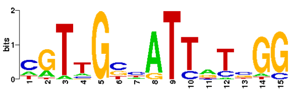	CGTTGnnATTnTn GG

The logo is for the best motif identified by MEME in each cluster.
